# Etiological Profile and Short-Term Outcomes of Acute Kidney Injury in Term Neonates at a Tertiary Care Centre in Western Maharashtra, India

**DOI:** 10.7759/cureus.66878

**Published:** 2024-08-14

**Authors:** Varsha Premkumar, Sudhir Malwade, Shailaja V Mane, Amulya Dharmagadda

**Affiliations:** 1 Pediatrics, Dr. D. Y. Patil Medical College, Hospital and Research Centre, Dr. D. Y. Patil Vidyapeeth (Deemed to be University), Pune, IND

**Keywords:** acute kidney injury, acute renal failure, kidney injury, mortality, etiology

## Abstract

Background

Acute kidney injury (AKI) is characterized by a sudden decline in kidney function, leading to a reduced glomerular filtration rate (GFR). This decline results in the accumulation of nitrogenous waste products in the blood, disturbs electrolyte balance, and disrupts fluid regulation.

Objective

To determine the etiological profile of AKI in term neonates.

Methods

A prospective observational study was conducted at the Neonatal Intensive Care Unit (NICU) of our tertiary care hospital and referral and teaching center. The study spanned a period of two years, from August 2022 to July 2024, and comprised a total of 78 term babies diagnosed with AKI, all of whom were enrolled after obtaining consent using a predefined proforma. The neonatal period was defined as the time from birth up to 44 weeks of postmenstrual age (PMA), encompassing a critical developmental phase in newborns.

Results

In our study of 78 term neonates with AKI, we found a predominant occurrence in males (53, 67.9%) and a significant proportion with low birth weights (41, 52.6%). The most common cause of AKI was sepsis or multiple organ dysfunction syndrome (MODS) (32, 41%), followed by perinatal hypoxia (14, 17.9%) and urinary tract obstructions (12, 15.3%). Urinary tract infections (UTIs) accounted for nine cases (11.5%), hypernatremic dehydration for six cases (7.6%), acute tubular necrosis for three cases (3.8%), and congenital polycystic kidney disease for two cases (2.9%). Mortality was notably high, with 20 neonates (25.7%) dying from AKI, particularly those with sepsis/MODS and perinatal hypoxia. However, conditions such as urinary tract obstructions and UTIs generally had better outcomes. The statistical analysis revealed a significant association between the underlying etiology and outcomes (p<0.001), underscoring the importance of prompt and targeted interventions for different AKI causes in neonates.

Conclusion

Our findings highlight the diverse etiological spectrum of AKI in term neonates and its significant impact on mortality. Early recognition, appropriate management, and targeted interventions tailored to the underlying cause are crucial in improving outcomes for neonates with AKI.

## Introduction

Acute kidney injury (AKI) is a clinical condition marked by a temporary buildup of urea, creatinine, and other nitrogenous waste products, along with disturbances in fluid and electrolyte balance [1]. The term "Acute Kidney Injury" was adopted to better reflect the spectrum of kidney dysfunction observed in clinical practice, as "failure" only represents a subset of the conditions. Furthermore, "kidney" has replaced "renal" to facilitate clearer communication with patients and their families, as the term "renal" is less commonly understood by the general public [2].

The prevalence of AKI varies among different populations. Approximately 5-12% of patients admitted to intensive care units exhibit different degrees of AKI [3,4]. Among critically ill children, mortality rates associated with AKI are notably high, ranging from 9% to 67% [5].

Neonatal AKI can arise from various insults occurring during the prenatal, perinatal, or postnatal periods. Prerenal AKI, the most common type in neonates, accounts for 85% of cases and typically results from decreased renal blood flow. This reduction can be caused by compromised placental blood flow (e.g., placental abruption), increased insensible losses (particularly in premature neonates with immature skin), and excessive gastrointestinal losses. Additionally, prerenal AKI may stem from decreased oncotic pressure due to hypoalbuminemia or increased capillary permeability in sepsis [6,7]. Renal AKI may occur due to vascular issues, such as bilateral renal vein thrombosis or renal artery thrombosis, which are often associated with malpositioned umbilical artery catheters or renal infarcts [6].

Forty to fifty percent of pediatric patients with AKI exhibited symptoms of chronic renal insufficiency. Prerenal injury, intrinsic renal disease (including vascular insults), obstructive uropathies, and prerenal injury are all subtypes of AKI. Ascertain the probable etiology of AKI by reviewing the patient's medical history, physical examination, and laboratory analyses, including urinalysis and radiographic findings. Many cases, including AKI in children who are hospitalized, are likely to have multiple factors at play in their etiology [8].

## Materials and methods

This prospective observational study was conducted in the NICU at Dr. D. Y. Patil Medical College from August 2022 to July 2024. Institutional ethics committee clearance was obtained before commencement, and written consent was taken from the parents or legal guardians of all term newborns.

Inclusion criteria

The study specifically included all term newborns who were admitted to the NICU. Neonates with congenital anomalies other than kidney and urinary tract anomalies, syndromic babies, and preterm babies born before 37 weeks of gestation were excluded.

Sample size

Based on the proportion of sepsis/multiorgan dysfunction and perinatal asphyxia in term neonates, estimated at 72.2%, with a 95% confidence interval and an acceptable difference of 7.5%, the sample size was calculated to be 78. The calculation was done using WinPepi Version 11.38.

Data collection and interpretation

Upon admission, detailed current and perinatal histories were collected. Radiological investigations, including antenatal sonography, were performed on neonates with anomalies. Baseline investigations included complete blood count, urea, creatinine, electrolytes, urinalysis, urine and blood cultures, and ultrasounds of the kidneys, ureters, and bladder. The samples were processed for immunoassays using the Abbott ARCHITECT C8000 system (Abbott Laboratories, Abbott Park, IL, USA). The diagnosis of AKI was established based on the pRIFLE (Pediatric Risk, Injury, Failure, Loss, End-Stage Renal Disease) criteria, with the estimated glomerular filtration rate (GFR) determined using the Schwartz equation [9]. Additional tests, such as complement 3, antinuclear antibody, anti-double-stranded DNA (dsDNA), and renal biopsy, were conducted as needed based on individual cases.

The normal renal parameters in term neonates are critical for identifying deviations that may suggest AKI. Healthy term newborns have a GFR between 15 and 60 mL/min/1.73 m². Serum creatinine level in healthy term neonates ranges from 0.75 mg/dL at birth to approximately 0.5 mg/dL by seven days of life. Serum urea levels in a newborn at 24 hours of life normally range from 3 to 12 mg/dL. These benchmarks provided a reference point for evaluating renal function in the neonates enrolled in this study.

Statistical analysis

The data were entered and analyzed using GraphPad Prism 10.0, Released July 2023 (Dotmatics, Boston, Massachusetts). Categorical variables are presented as frequency. A chi-square test was used to determine the association between the etiology and outcome of patients.

## Results

Among 78 term babies with AKI, the gender distribution among the participants was observed to be predominantly male, with 53 infants (67.9%) identified as male and 25 infants (32.1%) as female. The distribution of birth weights showed that the majority of infants, 41 out of 78 (52.6%), were categorized as having low birth weight, defined in this study as between 1 and 2.5 kg. The remaining 37 infants (47.4%) were classified as having normal birth weights, ranging from 2.6 to 3.5 kg.

Consanguinity can influence genetic predispositions and health outcomes in offspring, making it a relevant factor to consider. Within the study cohort, the majority of infants, 65 of 78 (83.3%), were born to parents who were not consanguineously related. There were 11 infants (14.1%) born to parents in a second-degree consanguineous marriage and two infants (2.6%) born to parents in a third-degree consanguineous marriage.

We analyzed the etiology of AKI in 78 term neonates. The most prevalent cause was sepsis/multiple organ dysfunction syndrome (MODS), accounting for 32 cases (41%). Perinatal hypoxia was the second most common cause, representing 14 cases (17.9%). Hydroureteric or posterior urethral valve obstructions accounted for 12 cases (15.3%). Urinary tract infections (UTIs) were found in nine cases (11.5%). Hypernatremic dehydration, which results from excessive sodium levels due to dehydration, was observed in six cases (7.6%). Less common causes included acute tubular necrosis, seen in three neonates (3.8%), and congenital polycystic kidney disease affected two (2.9%) cases (Table 1).

**Table 1:** Distribution of etiology in the participants. MODS: multiple organ dysfunction syndrome; UTI: urinary tract infection.

Etiology	Number (n=78)	Percentage
Congenital polycystic kidney disease	2	2.9
Sepsis/MODS	32	41
Hypernatremic dehydration	6	7.6
Hydroureteric/posterior urethral valve	12	15.3
Perinatal hypoxia	14	17.9
UTI	9	11.5
Acute tubular necrosis	3	3.8

The outcome analysis of 78 term neonates with AKI revealed a significant mortality rate, with 25.7% (20 neonates) succumbing to the condition. Conversely, the majority of the neonates, 74.3% (58 neonates), were successfully treated and discharged from the hospital.

**Figure 1:**
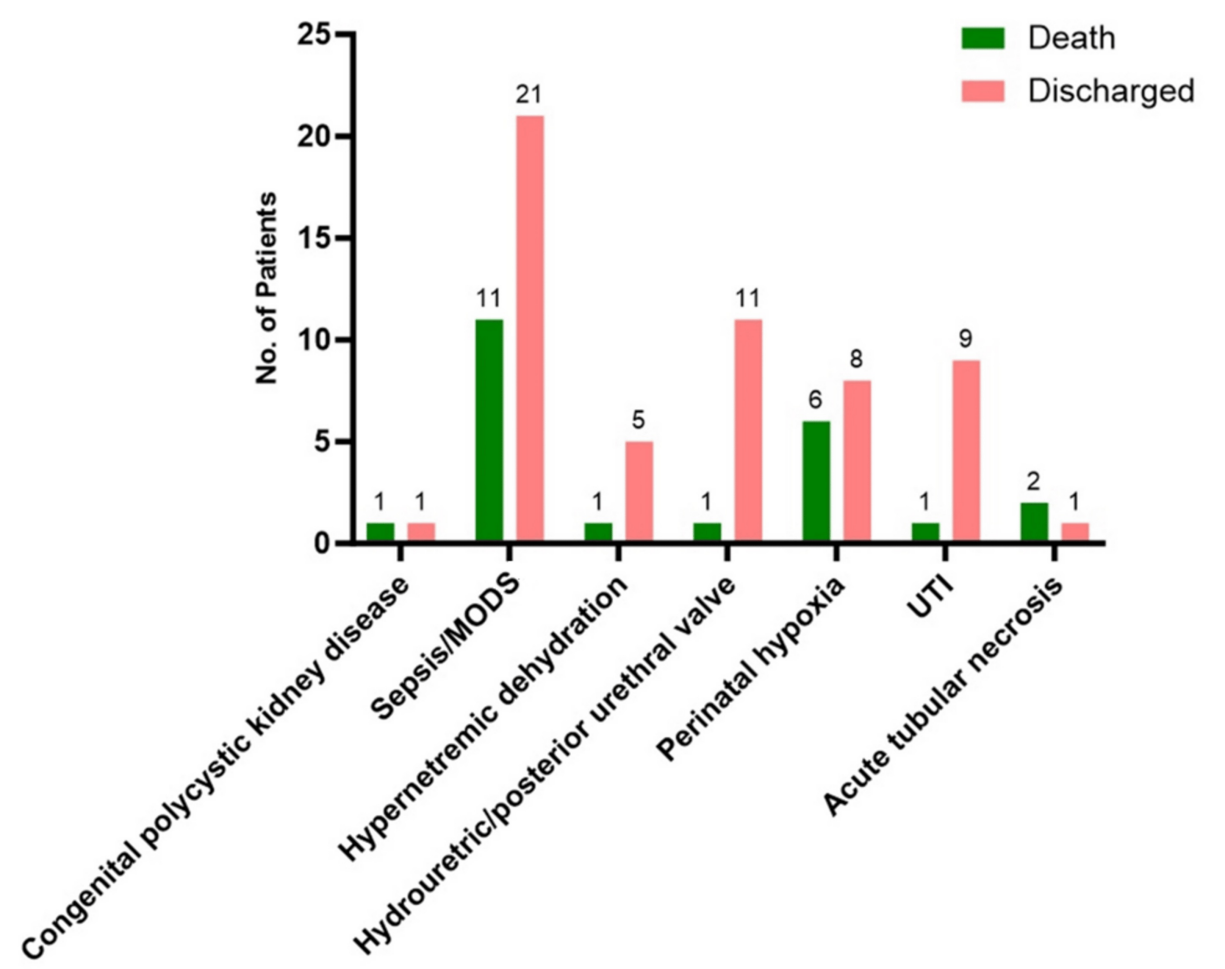
Association of etiology with the outcome of participants. MODS: multiple organ dysfunction syndrome; UTI: urinary tract infection.

We further analyzed the association between outcomes of term neonates with AKI and the underlying etiology. Congenital polycystic kidney disease, despite its rarity, led to one death and one discharge. Sepsis/MODs was the most critical condition, with 11 (34%) of 32 (41%) neonates succumbing to the condition, highlighting the severe impact of systemic infections and the need for prompt, aggressive treatment. Hypernatremic dehydration resulted in one death out of six cases, emphasizing the importance of timely correction of fluid and electrolyte imbalances. Urinary tract obstructions, such as hydroureteric or posterior urethral valve issues, had a favorable prognosis with 11 (92%) of 12 neonates discharged and one death. Perinatal hypoxia showed a high mortality rate, with six deaths (43%) of 14 cases. UTIs generally had positive outcomes, with nine (90%) of 10 cases resulting in discharge. Conversely, acute tubular necrosis had a poor prognosis, with two (7%) of three neonates not surviving, highlighting the severity of this condition. Overall, the p-value of less than 0.001 indicated a statistically significant association (Table 2, Figure 1).

**Table 2:** Association of etiology with outcome of the participants. MODS: multiple organ dysfunction syndrome; UTI: urinary tract infection. *Statistically significant.

Etiology	Outcome		Total	p-value
	Death	Discharged		
Congenital polycystic kidney disease	1	1	2	<0.001*
Sepsis/MODS	11	21	32	
Hypernatremic dehydration	1	5	6	
Hydroureteric/posterior urethral valve	1	11	12	
Perinatal hypoxia	6	8	14	
UTI	1	9	9	
Acute tubular necrosis	2	1	3	

## Discussion

AKI in neonates is increasingly recognized as a complication in hospitalized patients with acute illnesses [9]. The spectrum of AKI ranges widely, from minor changes in biochemical markers of kidney function to severe kidney failure that may require renal replacement therapy.

In our study, among the 78 term babies with AKI, the gender distribution was predominantly male, with 53 infants (67.9%) identified as male and 25 infants (32.1%) as female. Birth weight analysis revealed that 41 infants (52.6%) had low birth weights (1-2.5 kg), while 37 infants (47.4%) had normal birth weights (2.6-3.5 kg). Consanguinity was also considered, with 65 infants (83.3%) born to non-consanguineous parents, 11 infants (14.1%) to parents in a second-degree consanguineous marriage, and two infants (2.6%) to parents in a third-degree consanguineous marriage.

In our study, we observed that the etiology of AKI among neonates varied significantly, with 32 (41%) cases associated with sepsis or MODS. Among these cases, sepsis was predominantly early onset, leading to the death of 11 neonates (34%) while 21 (66%) were discharged. This high incidence and severity highlight the critical impact of sepsis on neonatal kidney function and overall outcomes. Congenital polycystic kidney disease was relatively rare, seen in only two cases (2.5%), and hypernatremic dehydration was identified in six neonates (7.6%). Perinatal hypoxia, another significant cause, was linked to six deaths (43%) and eight discharges (47%), underscoring its serious implications on neonatal health. Neonates with UTIs had a more favorable prognosis, with all cases resulting in discharge. However, acute tubular necrosis was particularly severe, with all three (100%) diagnosed neonates succumbing to the condition, illustrating its high mortality rate.

The findings of our study align with those reported in other research. For instance, Coggins et al. in a study revealed that 40 patients (20%) developed AKI shortly after evaluation of sepsis [10]. This underscores the critical relationship between sepsis and AKI development in neonates. Similarly, Tresa et al. identified various etiologies of AKI in children, noting that post-infectious glomerulonephritis (PIGN) and crescentic glomerulonephritis were common in primary renal cases, obstructive urolithiasis in postrenal cases, and sepsis in prerenal cases [11]. This broad spectrum of causes highlights the complexity and multifactorial nature of AKI in pediatric populations.

Further, Gallo et al. reported that perinatal asphyxia was the most common etiology of AKI in 72 term infants, accounting for 72.2% of cases, followed by congenital anomalies of the kidney and urinary tract (CAKUT) at 8.3%, congenital heart disease at 6.9%, and sepsis at 2.8% [12]. Bansal et al. observed through multivariate logistic regression that the male gender and sepsis were significant factors associated with AKI [13]. In contrast, Mohamed et al. found that infants with CAKUT frequently presented with severe AKI, particularly when there was bilateral kidney involvement, which was often diagnosed prenatally with associated oligo/anhydramnios [14].

These findings suggest regional variations in the etiology of AKI, with Western countries commonly reporting perinatal asphyxia and congenital anomalies, whereas sepsis is more frequently reported in Indian studies. This variation may reflect differences in healthcare practices, early diagnosis, and management strategies between regions.

One of the strengths of our study is its design as a prospective observational study, allowing for comprehensive data collection on various clinical and etiological factors associated with AKI in term neonates. This detailed approach provides valuable insights into the relationship between etiological factors and AKI outcomes. Furthermore, the study involved thorough investigation and follow-up of patients until discharge or death, which will contribute to the development of protocols and guidelines for the care of critically ill neonates, potentially enhancing outcomes through evidence-based practices.

However, there are limitations to consider. The study was conducted at a single tertiary teaching hospital, which means the sample size may not be representative of the general population. Additionally, the study could not explore predictors of mortality in AKI comprehensively, indicating a need for more extensive research. Despite these limitations, the prospective nature of the study provides relevant information about AKI among neonates encountered at a major referral center in a developing country.

## Conclusions

The study underscores the complex and varied causes of acute kidney injury (AKI) in term neonates, with significant implications for mortality. Early identification of AKI triggers, such as sepsis/MODs, perinatal hypoxia, and urinary tract obstructions, is pivotal for timely intervention. Tailored management strategies that address the specific underlying cause of AKI are essential for improving outcomes and reducing mortality rates, particularly in high-risk neonates. This approach not only emphasizes the importance of precision medicine in neonatal care but also highlights the need for ongoing research to refine diagnostic methods and therapeutic protocols aimed at mitigating the impact of AKI on this vulnerable population.
